# Intelligent medication manager: developing and implementing a mobile application based on WeChat

**DOI:** 10.3389/fphar.2023.1253770

**Published:** 2023-08-21

**Authors:** Jian Liu, Yalan Wu, Yuhua Wang, Pingfei Fang, Bikui Zhang, Min Zhang

**Affiliations:** ^1^ Department of Pharmacy, The Second Xiangya Hospital, Central South University, Changsha, China; ^2^ Institute of Clinical Pharmacy, Central South University, Changsha, China

**Keywords:** intelligent medication manager, WeChat app, medication guidance, medication consultation, medication reminder

## Abstract

**Background:** Time and space constraints have often hindered the provision of optimal pharmaceutical care, limiting medication therapy management. Social media tools have gained significant popularity in the field of pharmaceutical care. This study aimed to develop a WeChat-based intelligent medication manager platform that facilitates online pharmaceutical care and encourages self-management.

**Methods:** We developed a WeChat-based Internet pharmacy service platform called Xiang Medicine Guidance (XMG). Through the analysis of surveys and user access data, we evaluated the demand and utilization of the XMG platform and assessed patients’ satisfaction with its services. Patients’ adherence before and after the XMG platform intervention was also investigated.

**Results:** The XMG platform was launched in November 2022, offering medication guidance, reminders, and consultation services through the WeChat mini-program. By the end of April 2023, the platform had attracted 141.2 thousand users, accumulating 571.0 thousand visits. Moreover, 1,183 clients sought online medication consultations during this period. Six months after the launch of XMG, an impressive 91.02% of users expressed their satisfaction with the platform. The medication reminders and consultations provided by XMG significantly contributed to medication adherence, with 56.02% of users categorized as having good adherence, better than the previous 47.26%.

**Conclusion:** Through its services and features, XMG empowers patients to better manage their medications, seek professional advice, and adhere to their prescribed treatment plans. XMG has the potential to positively impact public health on a broader scale.

## 1 Introduction

The therapeutic efficacy of medications is closely related to patients’ adherence to drugs and the ability to self-manage their treatment. However, in China, there is a significant disparity between the number of licensed pharmacists and the population, leading to challenges in patient care. In February 2023, the China Food and Drug Administration reported only 716,207 registered pharmacists (5.1 per 10,000 people). This figure falls short of the global standard of 6.2 pharmacists per 10,000 population observed in developed countries (Feng et al., 2022). Furthermore, the distribution of pharmacy resources throughout China is uneven, with most pharmacists concentrated in teaching hospitals or urban areas. This leaves remote and impoverished regions with limited access to pharmacy services (Feng et al., 2022). The scarcity of pharmacists contributes to inefficiencies in the healthcare system. Pharmacists are burdened with repetitive dispensing tasks, which detracts from their valuable time. The time and space constraints associated with pharmaceutical care hinder the effective management of medication therapy, leading to various long-term medication-related issues (Zhang et al., 2020). For example, many patients do not know how to administer medications correctly. Some patients take drugs without fully understanding their potential adverse drug reactions (ADRs). After leaving the hospital, patients often lack avenues to consult a doctor or pharmacist for guidance. Recognizing these challenges, healthcare administrators have called hospitals to prioritize information infrastructure development, strengthen patient follow-up, and provide comprehensive medication guidance services.

Effective communication and trust between professionals are crucial to improving patient health outcomes (Zheng et al., 2022). Fortunately, the rapid advancement of information technology has expanded the scope of pharmaceutical services beyond traditional pharmacy dispensing processes and clinics (Yap et al., 2009; Xiao et al., 2013). Informatics and the internet have gained significant popularity in the modern pharmaceutical care system, offering a partial solution to the problem of insufficient pharmacists in remote areas (Wang et al., 2022). Various tools and platforms, including multimedia resources, self-service inquiry machines, and WeChat platforms, have been recommended to provide patients with valuable information on drug usage, dosage, and precautions.

Social media-based tools are increasingly tailored to specific needs to overcome the challenges posed by limited location-based resources and improve the speed of information sharing in healthcare systems (Redfern et al., 2013; Patel et al., 2015; Jalil et al., 2019; Wang et al., 2021). Recognizing the potential of these tools, the China Food and Drug Administration has emphasized the role of pharmacists in physical medical institutions in providing pharmaceutical care on the Internet. These pharmacists are tasked with offering online medication guidance, medication consultations, and other related services, focusing on patients with chronic conditions such as hypertension and diabetes. In China, pharmacists are exploring mobile health approaches using existing popular APPs to promote the rational use of medications among patients (Chen et al., 2020; Hua et al., 2020; Sun et al., 2020).

In China, WeChat is the dominant social media application, boasting an international monthly active user base of more than 1.268 billion in 2021 (Tso, 2022). Globally, WeChat is among the most popular social networks, along with WhatsApp, Facebook, YouTube, and Instagram (Li et al., 2019a). Although WeChat’s user base and influence continue to grow, its application and adoption among healthcare professionals have received relatively less attention. In recent years, particularly with the spread of COVID-19, WeChat has been used in various healthcare settings to improve patient care in China (Hua et al., 2020; Li et al., 2021; Liang et al., 2022). Physicians, nurses, and pharmacists have embraced the innovative application of WeChat to enhance patient comprehension of treatment and improve follow-up management. Notable examples include the implementation of a self-management intervention through WeChat, as demonstrated in a randomized controlled trial conducted in Guangzhou province (Li et al., 2019b). The trial showed that a 6-month health education intervention, health promotion, group chat, and blood pressure monitoring via WeChat helped patients lower their blood pressure and improve self-management effectiveness. Another study in Shandong province confirmed the positive impact of a WeChat-based pharmaceutical care program in post-discharge patients with non-insulin-dependent diabetes mellitus and hypertension. This program markedly improved medication adherence among participants (Wang et al., 2022). As a leading Internet messaging tool, the multifunctional WeChat official account and WeChat groups have effectively served as a communication bridge between patients and medical staff, leading to successful implementation in disease-specific pharmaceutical care. However, the current approach of increasing communication within a limited number of patient groups is no longer sufficient to meet the growing patient demand.

The medical landscape has witnessed the proliferation of more than 318,000 medical apps designed to aid in disease diagnosis and management (Huang et al., 2022). One such notable example is the novel shared WeChat app developed by Huang et al., specifically tailored to address dental anxiety. This app has demonstrated its effectiveness in providing support before and after dental procedures, and in assisting the management of high-risk patients during the COVID-19 pandemic. Beyond dental anxiety, private healthcare support companies have used WeChat capabilities to improve the monitoring and collection of personal health data (Zhang et al., 2017; Tso, 2022). Despite these advances, there remains an urgent need to develop a comprehensive pharmaceutical care app that caters to the needs of all outpatients.

To promote high-quality pharmaceutical care and advance the construction of a healthy China, the Second Xiangya Hospital of Central South University in Changsha, China, has established a WeChat-supported platform called Xiang Medicine Guidance (XMG). This platform allows patients to access comprehensive medication guidance, medication reminders, and online pharmaceutical consultation services. This study aimed to create a WeChat-based intelligent medication manager platform that facilitates pharmaceutical care and promotes self-management. The impact of this platform was assessed through patient surveys and analysis of platform access.

## 2 Materials and methods

### 2.1 Study design

This study consisted of two surveys. The initial survey was conducted before the launch of XMG to gather data on patients’ perceptions of medication guidance. The survey aimed to assess the perceived necessity of medication guidance, how patients currently receive medication guidance, the specific content of medication guidance they desired, and patients’ medication adherence. The questionnaire survey form is shown in Supplementary Table S1. The information obtained from this survey played a crucial role in the design and development of XMG. The survey participants were randomly selected from patients visiting outpatient clinical departments at the Second Xiangya Hospital of Central South University between January and March 2022. In April 2023, 6 months after the launch of XMG, a second survey was conducted among users of the XMG platform to evaluate the impact of XMG, including user satisfaction with the platform and its influence on medication adherence. These users included individuals who independently accessed XMG or accessed it with the help of their relatives on smartphones. The survey specifically targeted XMG users who visited outpatient clinical departments. The questionnaire survey form is listed in Supplementary Table S2.

The sample size (n) was calculated using the following formula to estimate the proportion of a single population: *n* = (Z_α/2_)^2^p (1-p)/d^2^. With a 95% confidence interval and a 5% marginal error, the minimum sample size required was determined as follows: n = (1.96)^2^ * (0.5) * (0.5)/(0.05)^2^ = 384. Since no published studies were comparable to this study, a proportion value (p) of 0.5 was used in the calculation. A sample size greater than 384 was considered scientific and feasible. To account for the expected non-response rate, 10% was added to the minimum sample size, and the final sample size was 422.

The following question was asked to assess medication adherence: “Do you experience instances of missed doses or non-adherence with medication instructions?” The response options were as follows: Frequently, Occasionally, Rarely, and Never. If the response was frequent or occasionally, it was categorized as poor adherence. Otherwise, if the response was rare or never, it was classified as good adherence.

The inclusion criteria for both surveys required participants to be able to speak and write Mandarin Chinese. Patients who did not wish to participate were excluded. The questionnaires were administered and completed on-site. The questionnaires collected demographic and socioeconomic data, including information on sex, age, marital status, educational background, place of residence, and number of medical visits.

In addition to the questionnaire survey, the impact of the XMG platform was evaluated by analyzing user access data. Various metrics were considered, including the duration of access, the number of clicks made, the number of pages visited, the types of medication consultations sought, and the outpatient clinical departments visited by patients.

### 2.2 The development of the WeChat-based intelligent medication manager

XMG is a smartphone WeChat APP that has been collaboratively designed and developed by Beijing Zuoyi Technology Co., LTD. and the Department of Pharmacy at the Second Xiangya Hospital of Central South University. XMG was officially launched in November 2022. This integration was embedded in the WeChat network platform, enabling comprehensive pharmaceutical care services. The underlying intelligent algorithm technology encompassed various components, such as large-scale text mining, structured data-based knowledge graph construction, intelligent interaction, and an intelligent disease diagnosis system. The knowledge base incorporated many authoritative sources, including the China Pharmacopeia, drug instructions, clinical treatment guidelines, and reputable medical books. The algorithm learning process involved analyzing a substantial amount of medical papers, popular science articles, and the expertise of physicians and pharmacists. Furthermore, recognizing the importance of customized interaction, a suitable intelligent interactive network system was developed for pharmacists and patients, considering the hospital’s unique circumstances.

### 2.3 The content of the WeChat-based intelligent medication manager

**FIGURE 1:**
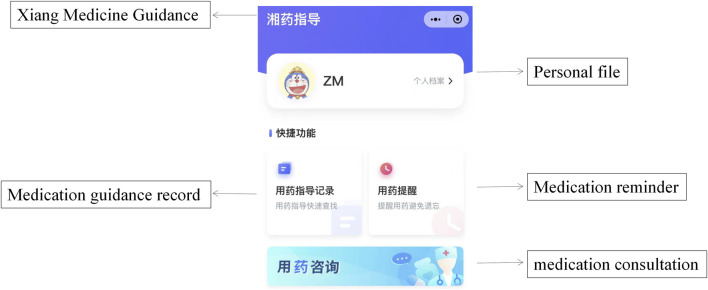
The main interface of the Xiang Medicine Guidance provides convenient access to three essential sections. Medication Guidance, Medication Reminder, and Medication Consultation. Patients can easily find and use the Xiang Medicine Guidance mini program by simply scrolling down on the main screen of WeChat, without the need to download additional applications.

**FIGURE 2:**
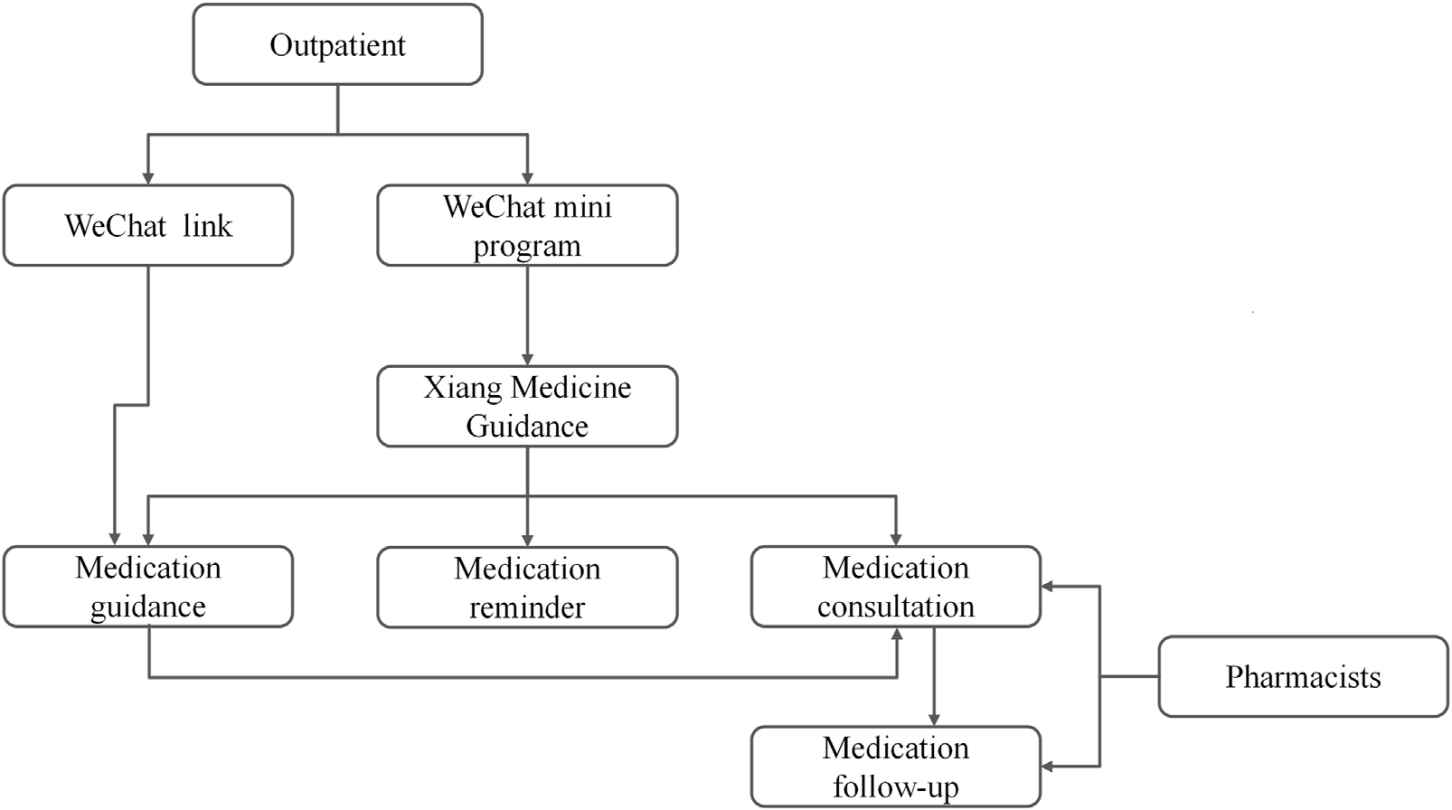
The Xiang Medicine Guidance pharmacy service mode.

The hospital’s outpatient pharmaceutical care system is integrated into a cloud database named XMG (Figure 1), which provides patients with convenient access to medication guidance, medication reminders, and medication consultation through a WeChat mini program, eliminating the need to download additional applications. Figure 2 shows the XMG pharmacy service framework. Once a drug is prescribed, patients receive an automated medication guidance report from the hospital’s official WeChat account. Clicking the message link allows patients to access the medication guidance report, review medication reminders, and interact online with senior pharmacists. XMG has the following three interfaces.

#### 2.3.1 Medication guidance interface

**FIGURE 3:**
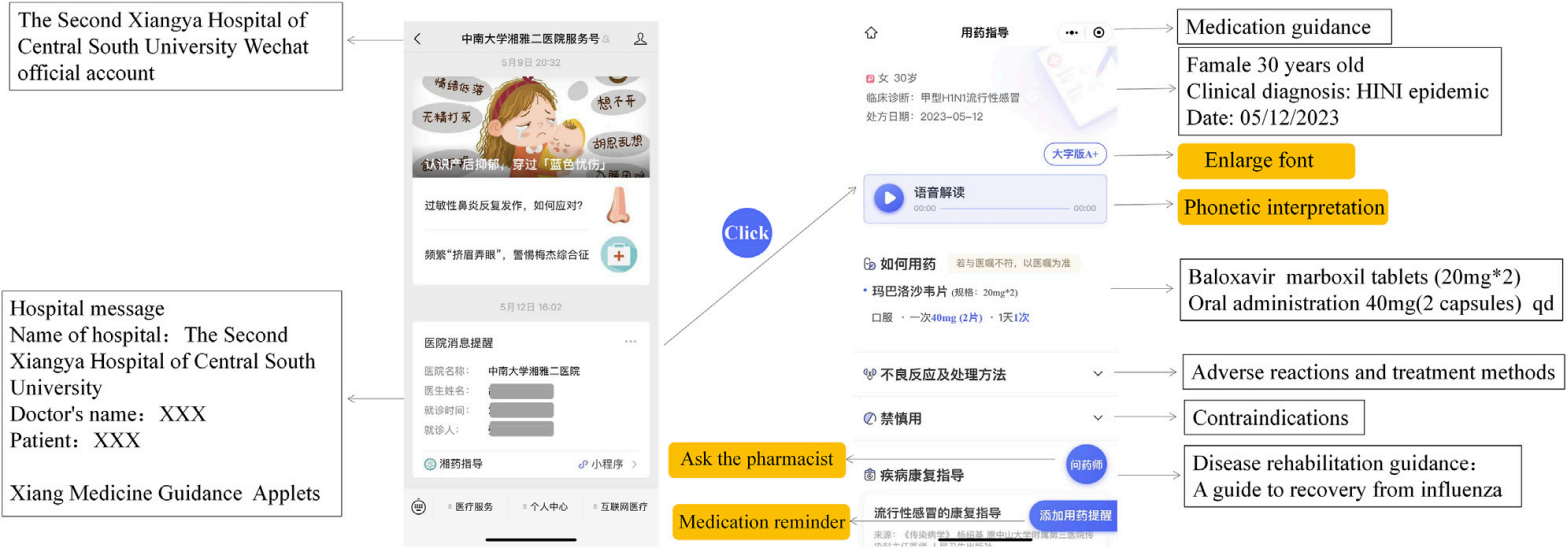
Medication guidance interface. After prescribing, a drug guidance report will be sent to patients’ WeChat, through which, patients can enter the interface of medication reminder and medication consultation interface.

After prescribing medications, patients will receive a drug guide report through their WeChat accounts (Figure 3). This comprehensive report includes essential information such as dosage and administration instructions, precautions, warnings, potential ADRs, contraindications, and popular science education services like disease rehabilitation guidance and healthy lifestyle recommendations. To accommodate individual needs and preferences, personalized settings are encouraged within XMG. For patients with visual impairments or limited literacy, XMG offers a voice function that allows medication instructions to be spoken for ease of comprehension. Additionally, XMG supports automatic font enlargement for older patients to enhance readability. Furthermore, XMG converts the units of measurement on the prescription into easily understandable packaging units, such as “one tablet” or “one capsule.”

#### 2.3.2 Medication reminder interface

**FIGURE 4:**
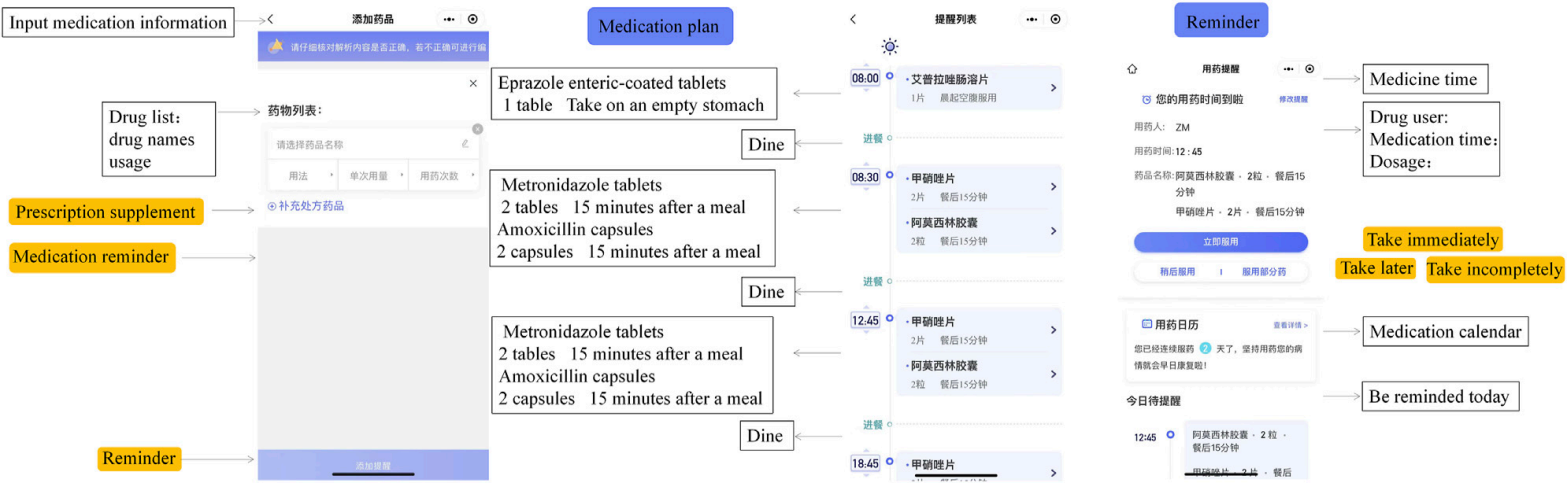
Medication reminder interface. Patients can add a medication plan and enjoy an alarm clock reminder service.

XMG incorporates intelligent computing to determine the optimal time for patients to take each medication, considering factors such as drug absorption, patient daily habits, and potential medication interactions (Figure 4). When it is time for patients to take their medications, XMG provides a reminder alarm or notification. For drugs with a limited course of treatment, XMG reminds patients to discontinue the medication at the appropriate time. Similarly, for drugs with a limited shelf life after opening, such as eye drops, XMG notifies patients when the drug is no longer suitable for use. In XMG, if patients record the remaining doses of their medications in the “My Medicine Cabinet,” they will receive timely reminders when the quantity of the drug is running low. These reminders prompt patients to schedule a visit to the hospital for a refill or prescription renewal.

#### 2.3.3 Medication consultation interface

**FIGURE 5:**
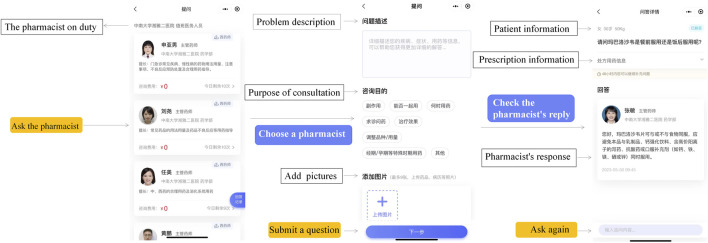
Medication consultation interface. When having medication questions, patients can consult the pharmacist on duty through text, pictures, and prescription.

XMG offers convenient online medication consultations to patients. At any time and from anywhere, patients can access the “Ask Pharmacist” feature within XMG to ask questions about their medications. They can communicate by texting or sharing pictures (Figure 5). Pharmacists are available to provide one-to-one online medication consultation services to patients. These consultations can be conducted through text or voice interactions depending on the pharmacists’ preference.

Furthermore, pharmacists can use XMG to follow up with patients. The patients will be systematically screened through the hospital information system when the follow-up plan is formulated. The XMG platform will regularly send popular science education messages and medication follow-up questionnaires to specific patients. These inquiries may cover medication adherence, efficacy, and ADRs. Pharmacists or physicians can then evaluate the patient’s treatment progress and engage in real-time communication with the patient based on the collected information. For patients with poor medication adherence, unsatisfactory drug treatment effect, or severe ADRs, the pharmacist will contact them by telephone and guide them to use the drug rationally.

### 2.4 Data analysis

Data were analyzed using SPSS software (version 22.0, SPSS Inc., Chicago, United States). Categorical variables are expressed as numbers and percentages.

## 3 Results

### 3.1 Patient characteristics

In the first survey, 450 questionnaires were distributed, and 419 valid questionnaires were analyzed. The demographic and clinical characteristics of the patients are presented in Table 1. Most patients were under 35 years of age (51.07%) and had a bachelor’s degree as their highest level of education (57.56%). Most patients resided in urban areas (65.39%).

**TABLE 1:** Demographic and clinical characteristics of the patients (*n* = 419).

Variable	Number (proportion)
Sex	
Male	213 (50.84%)
Female	206 (49.16)
Age, years	
<35	214 (51.07%)
35–49	104 (24.82)
50–64	73 (17.42%)
≥65	28 (6.68%)
Marital status	
Married	207 (49.40%)
Separated/divorced/widowed	212 (50.60%)
Educational background	
High school or below	150 (35.80%)
Bachelor degree	242 (57.56%)
Postgraduate degree or above	27 (6.44%)
Residence	
Urban	274 (65.39%)
Rural area	145 (34.61)
Number of medical visits	
1	171 (40.81%)
2	145 (34.61%)
≥3	103 (24.58%)

The need and patient requirements for pharmaceutical care are summarized in Supplementary Table S3. Although 98.33% of patients considered medication guidance necessary, only 17.42% consulted pharmacists when they had questions about their medications. Regarding the preferred content and format of medication guidance, 45.83% preferred Internet or telephone-based consultations. Furthermore, 78.28% wanted medication reminder services through a mini program. Among the various aspects of medication guidance, the dosage and administration of drugs were the most preferred (72.79%), followed by contraindications and precautions (65.63%) and indications (64.68%). As for medication adherence, only 47.26% of the users were considered to have good adherence.

### 3.2 User access data analysis

At the end of April 2023, XMG had attracted 141,200 users, with a remarkable 571,000 visits recorded over the 6 months. To examine the extent of patient engagement with the medication guidance report, we analyzed the length of visits, the number of clicks, and the number of pages accessed by clients in April 2023. The findings are presented in Table 2. The analysis revealed that 29.96% of the patients spent more than 100 s accessing the medication guidance report. Regarding the frequency of accessing the report, 44.31% of the patients opened it only once, while 20% accessed it twice. Regarding the number of pages visited within the report, 39.14% of the patients explored two pages.

**TABLE 2:** The analysis of user access to the “Xiang Medicine Guidance” in April 2023 (*n* = 36946).

Item	Number of visits	Proportion (%)
Access duration		
0–2 s	594	1.61
3–5 s	1753	4.74
6–10 s	2,739	7.41
11–20 s	4,627	12.52
21–30 s	3,864	10.46
31–50 s	5,472	14.81
51–100 s	6,828	18.48
>100 s	11,069	29.96
Number of clicks, times		
1	16,371	44.31
2	7,410	20.06
3	3,748	10.14
4	2,494	6.75
5	1,585	4.29
6–7	1,922	5.20
8–10	1,434	3.88
11–20	1,459	3.95
21–30	319	0.86
>30	204	0.55
Number of pages visited		
1	2,527	6.84
2	14,461	39.14
3	4,146	11.22
4	4,794	12.98
5	2,142	5.80
6–10	5,819	15.75
>10	3,057	8.27

Our medication consultation platform currently provides free pharmaceutical services for patients and the public to improve their awareness and satisfaction with pharmacists. Ten senior pharmacists were responsible for providing online medication consultation services. When a patient seeks consultation, the system automatically extracts relevant information such as the patient’s sex, age, doctor’s diagnosis, and prescription details. Pharmacists were expected to respond to patients within 12 h, and the completion rate for timely responses was 100%. Pharmacists answered 1,183 questions.

The data from these medication consultations were analyzed and summarized. Table 3 shows the demographic and clinical characteristics of patients seeking consultations. Among these consultations, 668 (56.47%) were from female patients. Most consultations (53.00%) were from patients under 35 years of age. Table 4 shows the content of the consultations, Table 5 displays the classification distribution of advice provided by pharmacists, and Table 6 shows the distribution of consultations across different departments. The five main categories of consultations requested by patients were ADRs (29.59%), dosage and administration (25.61%), drug-drug interactions (8.2%), treatment duration (6.68%), and contraindications (5.07%). Pharmacists informed the patients of the correct usage and dosage, common ADRs and their treatment, detailed medical procedures, whether to take drug and whether certain drugs can be taken together. Patients who visited the departments of psychiatry, obstetrics and gynecology, gastroenterology, dermatology, and the Internet hospital made the highest number of drug consultation inquiries. Table 7 provides several specific examples of consultations.

**TABLE 3:** Demographics of patients seeking consultations using “Xiang Medicine Guidance” (*n* = 1183).

Variable	Number of consultations	Proportion (%)
Gender		
Male	515	43.53
Female	668	56.47
Age, years		
<35	627	53.00
35–49	284	24.01
50–64	185	15.64
≥65	87	7.35

**TABLE 4:** The classification distribution of consultation contents (*n* = 1183).

Item	Number of consultations	Proportion (%)
ADRs	350	29.59
Dosage and administration	303	25.61
Drug-drug interactions	97	8.20
Course of treatment	79	6.68
Contraindications	60	5.07
Indications	46	3.89
Clinical curative effect	21	1.78
Medication reconciliation	20	1.69
Warning	15	1.27
Storage	12	1.01
Decocting method	10	0.85
Others	170	14.37

**TABLE 5:** The classification distribution of advice provided by pharmacists (*n* = 1183).

Item	Number	Proportion (%)
Rational use of medication	314	26.54
Common ADRs and their treatment	308	26.04
Detailed medical procedures	108	9.13
Whether the drug can be taken	93	7.86
Whether certain drugs can be taken together	82	6.93
Appropriate course of drug treatment	65	5.49
Mechanism of action and indications of drugs	43	3.63
Drug regimen optimization	37	3.13
Patient referred to prescriber	30	2.54
The healthy lifestyle	21	1.78
Precautions for taking medicine	18	1.52
Time of onset of the drug	16	1.35
Correct storage conditions	13	1.10
Treatment of drug misuse or overdose	13	1.10
Others	12	1.01
Correct preparation of traditional Chinese medicine	10	0.85

**TABLE 6:** Department distribution of patients seeking consultations (*n* = 1183).

Item	Number of patients	Proportion (%)
Department of psychiatric	326	27.56
Department of obstetrics and gynecology	93	7.86
Department of gastroenterology	92	7.78
Department of dermatology	89	7.52
Internet hospital	82	6.93
Department of urology	57	4.82
Department of cardiovascular medicine	46	3.89
Department of nephrology	42	3.55
Department of otolaryngology	39	3.30
Department of metabolism and endocrinology	36	3.04
Department of Traditional Chinese medicine	35	2.96
Department of neurology	35	2.96
Department of ophthalmology	26	2.20
Others	185	15.64

**TABLE 7:** Representative examples of the consultations on “Xiang Medicine Guidance.”

Content	Case 1	Case 2	Case 3	Case 4	Case 5	Case 6
Patient	Female, 66 years old	Female, 19 years old	Female, 19 years old	Male, 59 years old	Male, 40 years old	Female, 44 years old
Question	The prescription dose of leflunomide is 2 tablets 4 times a day, which exceeds the recommended dose	Adverse reactions after accidental intake of trazodone 100 mg: dizziness, dry mouth, fatigue	Can vitamin A palmitate eye gel be kept out of the refrigerator?	Can azilsartan tablets combining with doxazosin mesylate extended-release tablets cause low blood pressure?	May I take cefaclor capsules and metronidazole tablets after drinking alcohol yesterday?	How long should I take bacterial lysates capsules?
Disease	Rheumatoid arthritis	Anxiety-depressive state	Corneal layer change	Hypertension, prostatic hyperplasia	Acute periapical inflammation	Bronchiectasis
Current medications	Leflunomide tablets (20 mg qid); Celecoxib capsules (0.2 g bid); Lguratimod tablets (25 mg qd)	Trazodone hydrochloride tablets (50 mg qn)	Vitamin A palmitate eye gel (0.2 g bid); Azithromycin tablet (500 mg qd)	Azilsartan tablet (40 mg qd); Doxazosin mesylate extended-release tablet (4 mg qn)	Cefaclor capsule (500 mg bid); Metronidazole tablet (200 mg tid)	Citafloxacin capsule (100 mg bid); Acetylcysteine effervescent tablet (600 mg bid); Bacterial lysates capsule (7 mg qd)
Assessment	The frequency of leflunomide is incorrect. Contact the prescribing doctor to confirm that the frequency is qd	The maximum dose of trazodone for outpatient patients can reach 400 mg. The common adverse reactions are dry mouth, dizziness and fatigue	Vitamin A palmitate eye gel should be stored in the refrigerator before opening (2°C–8°C). After opening, the product can be stabilized at 15°C–25°C for 30 days	Doxazosin extended-release tablet is an alpha receptor antagonist that can be used for prostatic hyperplasia and hypertension	Cefaclor is a cephalosporin drug that does not contain the methiontetrazolium group. Alcohol consumption will not cause disulfide reaction. Nitroimidazole metronidazole can induce disulfiram reaction	For prophylactic, bacterial lysates capsules should be used for 10 days, then stop for 20 days. One course of treatment is 3 months. For adjuvant therapy, it should be used until symptoms disappear (at least 10 days)
Suggestion	The correct frequency of leflunomide tablets is qd	Taking 100 mg for the first time can cause dry mouth, dizziness, fatigue, and other adverse reactions, which is generally not life-threatening and requires no special treatment. Please take the medicine correctly	The product should be stored in the refrigerator before opening (2°C–8°C) and store at 15°C–25°C for 30 days after opening	Doxazosin can be used for hypertension. Thus, combining with azilsartan may cause hypotension. It is recommended to adjust the dosage of azisartan to 20 mg and monitor blood pressure closely	Metronidazole can cause disulfiram reaction, thus alcohol should not be consumed during treatment. Patients are advised to take medication 3 days after drinking alcohol	Inform the patient that bacterial lysate capsules should be used until symptoms disappear (at least 10 days) for adjuvant therapy
Classification	Dosage and administration	Adverse drug reactions	Storage	Drug-drug interactions	Others	Course of treatment

### 3.3 Patient satisfaction and medication adherence

In the second survey, 420 questionnaires were distributed, and 407 were considered valid and analyzed. Demographic data are shown in Supplementary Table S4, and patients’ satisfaction and medication adherence are exhibited in Supplementary Table S5. Users showed high satisfaction with XMG, with 91.40% (372 out of 419) expressing their satisfaction. In terms of medication adherence, 56.02% of the users were considered to have good adherence.

## 4 Discussion

Studies have reported that 50% of patients struggle to use medications properly (Brown et al., 2016). To address this problem, various strategies, such as adverse reaction monitoring, medication tracking, medication reminders, and recording of patient’s medication history, have been implemented to reduce medication errors. However, due to the shortage of professional pharmacists in China, providing high-quality pharmaceutical care has become a challenge. The traditional face-to-face pharmaceutical care model needs to be revised in response to this challenge. Consequently, many medical institutions and computer scientists use technology to offer patients more efficient and convenient healthcare services.

Several mobile health tools have been developed and researched to support patients in managing their medications. For example, a web-based pharmaceutical care plan application potentially facilitated the collaboration between healthcare providers and patients. In addition, Zhang et al. (2022) developed an anticoagulation management model for anticoagulation therapy in atrial fibrillation patients. This model aims to enhance rationality, adherence, and satisfaction for medical professionals and patients. Another example is the WeChat-supported platform called Medication Housekeeper, which promotes self-management of cancer patients experiencing pain by facilitating collaboration between physicians and pharmacists. This collaboration aims to optimize therapeutic outcomes (Zhang et al., 2021). However, most of these applications focus on managing drug therapy for specific medications or diseases, none provides comprehensive pharmaceutical care for outpatients with various medical conditions. We have designed and developed the XMG medication manager platform to provide outpatient medication guidance, reminders, and consultations.

The XMG has the following innovations. First, a localized deployment of servers where all patient prescription data is stored on a local server. Through a front-end device, we interact with a third-party company’s cloud-based knowledge repository and mobile applications, ensuring the privacy and security of patient data. This setup allows us to fully retain the rights to independently utilize research data and related intellectual property. Second, existing APPs provide medication guidance and consultation for specific groups (such as a particular disease or medication). However, XMG provides comprehensive online pharmaceutical services to all patients receiving treatment in our outpatient clinics and through Internet hospitals. Third, we proactively send medication guidance information to all outpatient patients via WeChat. The patient’s prescription data automatically accompany this information and can be used to enable medication reminders in one click. Patients with medication questions can easily access pharmacist consultations in one click. Fourth, patients who do not receive treatment at our hospital or individuals in the general public can also utilize the XMG mini-programs for online pharmaceutical consultations and medication reminders.

Implementing XMG can enhance pharmaceutical care in hospitals and improve the quality of medication management through information technology.

Numerous users have experienced the substantial impact of XMG in providing medication guidance and consultation, effectively supporting patients with their medication needs. The survey also indicates high user satisfaction with the pharmaceutical care provided by XMG. According to the report, multitudinous APPs only involved dozens or hundreds of patients for the comparative study; their number of users was not shown (Huang et al., 2022; Liu et al., 2022; Sun et al., 2022). The “Cloud Pharmacy Care” platform acquired 1,432 views and completed 39 counseling cases in 2 months, was much less than the XMG (Li et al., 2021). Despite the short establishment time, the patient’s attention and the number of consultations in XMG were very high. This may be mainly due to the significant number of outpatient clinics in our hospitals and the initiative of medication guidance information.

The availability of medication reminders and pharmacist consultations has played a crucial role in improving medication adherence. As a result, the proportion of users categorized as having good adherence increased from 47.26% to 56.02%. These results underscore the effectiveness of XMG in promoting medication adherence and enhancing patient outcomes. However, compared to other projects dedicated to improving the pharmaceutical management of a particular type of patient, XMG has a limited effect on patients’ medication adherence (Chen et al., 2020; Wang et al., 2022). It is closely related to the short establishment time and the lack of targeted patient medication management. In the next stage, the pharmacist would employ the medication follow-up function to conduct follow-up management for patients with specific diseases to improve their medication adherence. Through the evaluation of patient consultation records, several interesting findings have emerged. Women were more inclined to use XMG for medication counseling than their male counterparts, which was similar to that of other mini-program users (Li et al., 2021). Counseling was available for patients or the public of all ages. Despite efforts to simplify the mini program, online medication counseling still predominantly attracts patients under 35 years of age. This may be related to their willingness to accept new things.

The ADRs and the psychiatric department ranked first by analyzing the classification and department distribution. There may be several reasons for the difference between the literature (Gotlib et al., 2017; Patel et al., 2020). First of all, the reputation of our hospital’s psychiatric department is prominent in China, and many patients come here. Second, patients with psychiatric conditions are generally young and more able to use XMG. Most importantly, antipsychotic medications often cause ADRs, such as drowsiness, dizziness, hyperactivity, and sedation. This poses a significant obstacle to adherence to antipsychotic regimens. The responses and encouragement of pharmacists can play a crucial role in improving treatment outcomes for psychiatric patients. A significant challenge facing many patients is assessing, differentiating, and managing adverse drug reactions, particularly among psychiatric patients. In future work, we plan to follow patients with mental illness to improve their medication adherence and therapeutic effect. Consultations on using polyethylene glycol-containing electrolyte powder appropriately before colonoscopies and topical medications were common among patients. This highlights the importance of conducting in-depth medication education and counseling for these specific medications to ensure their proper use in the future.

In China, “Mobile Internet + pharmaceutical care” gradually changed medical practices and processes. XMG offered a solution to the problem of gaps in pharmaceutical management after patients left the hospital. Through XMG, patients can access and review the medication guidance report anytime, and healthcare professionals can efficiently monitor patient indicators and provide prompt suggestions. Furthermore, medication guidance collected from various hospitals can be consolidated into a comprehensive medication history, facilitating physicians’ understanding of a patient’s past medication experiences. The survey results demonstrate that XMG is a convenient, cost-effective, and reliable pharmacy application. Its benefits extend to outpatients and emergency cases, patients with chronic diseases, and people seeking daily pharmacy consultations.

This study has several limitations. First, the participants were recruited from a single tertiary hospital, which may restrict the generalizability of the findings to a broader population. Second, the study did not evaluate the impact of the intervention and medication adherence on clinical outcomes. Third, integrating patient information from the hospital information system into the XMG platform relies on the prescription number, which means that patients with multiple prescriptions from different doctors may receive multiple sets of medication instructions. In contrast, the consulting pharmacist can only access a single prescription, making it difficult to comprehensively evaluate the patient’s overall drug therapy regimen. Future efforts should focus on integrating medication and prescription data from hospital information systems to ensure a more comprehensive and accurate assessment. However, despite these limitations, the online medication guidance, reminder, and consultation framework has been successfully established and can serve as a foundation for the further development of paid pharmaceutical care services. With continued improvements and advancements, the platform can potentially enhance the provision of pharmaceutical care to patients.

## 5 Conclusion

An online pharmacy service platform (XMG) was developed to address patients’ medication-related issues. The XMG platform offers patients timely online access to interactive medication guidance, reminders, and consultations, effectively bridging the gap in medication management after hospitalization. With its personalized and intelligent features, XMG has the potential to revolutionize the way patients engage with their medications, leading to better health outcomes and improved patient experiences.

## Data Availability

The original contributions presented in the study are included in the article/Supplementary Material, further inquiries can be directed to the corresponding author.
