# Supplementary material for: Intelligent medication manager: developing and implementing a mobile application based on WeChat
Source: Front Pharmacol. 2023 Aug 21;14:1253770. doi: 10.3389/fphar.2023.1253770 (PMC10475577; doi:10.3389/fphar.2023.1253770)
Supplement: Supplementary file 1 [file Table1.DOCX]

**Questionnaire 1**

**Patient characteristics**

1. Your sex

🞎 Male

🞎 Female

2. Your age

🞎 < 35 years old

🞎 35-49 years old

🞎 50-64 years old

🞎 ≥65 years old

3. Your marital status

🞎 Single

🞎 Married

🞎 Divorced

🞎 Other

4. Your educational status

🞎 High school or below

🞎 College degree or bachelor's degree

🞎 Master's degree or above

5. Where do you live?

🞎 Town

🞎 Country

1. Number of previous outpatient services at the present facility

🞎 One

🞎 Two

🞎 More than two

1. The department in which you were treated

🞎 Internal medicine department

🞎 Surgery department

🞎 Gynecology and obstetrics department

🞎 Pediatric department

🞎 Psychiatry department

🞎 Others

**Patient attitude towards medication guidance**

8. What do you think about the medication guidance?

🞎 Very necessary

🞎 Generally necessary

🞎 Not necessary

9. What would you prefer when you are in doubt about medication?

🞎 Ask the doctor

🞎 Ask the pharmacist

🞎 Read the instructions

🞎 Ask a friend

🞎 Search on the internet

1. What will you do if your doctor's prescription does not conform to the instructions?

🞎 Take medication according to the doctor

🞎 Take medication according to the instructions

🞎 Consult the doctor again

🞎 Consult the pharmacist

11. Please indicate your level of satisfaction with the current medication guidance of our hospital

🞎 Very satisfied

🞎 Moderately satisfied

🞎 Not at all satisfied

**Outpatients' demand for medication guidance**

12. Please indicate the drug-related information you wanted from the hospital pharmacist

|  | Extremely needed | Moderately needed | No need |
| --- | --- | --- | --- |
| Indications | 🞎 | 🞎 | 🞎 |
| Dosage and administration | 🞎 | 🞎 | 🞎 |
| Contraindications and precautions | 🞎 | 🞎 | 🞎 |
| Adverse drug reactions | 🞎 | 🞎 | 🞎 |
| Drug-drug interaction | 🞎 | 🞎 | 🞎 |
| Storage | 🞎 | 🞎 | 🞎 |
| Expiration date | 🞎 | 🞎 | 🞎 |

1. Please indicate what information you wanted about dosage and administration?

|  | Extremely needed | Moderately needed | No need |
| --- | --- | --- | --- |
| Route of administration | 🞎 | 🞎 | 🞎 |
| Duration of medication | 🞎 | 🞎 | 🞎 |
| Sequence of drug use | 🞎 | 🞎 | 🞎 |
| Dose and interval of administration | 🞎 | 🞎 | 🞎 |
| The use of external drugs | 🞎 | 🞎 | 🞎 |

1. Please indicate the information you wanted about contraindications and precautions?

|  | Extremely needed | Moderately needed | No need |
| --- | --- | --- | --- |
| Dietary taboo | 🞎 | 🞎 | 🞎 |
| Normal reaction that may occur after medication | 🞎 | 🞎 | 🞎 |
| The effects of drugs on pregnant women and children | 🞎 | 🞎 | 🞎 |
| Influence on patients with allergies and idiosyncrasies | 🞎 | 🞎 | 🞎 |
| Effects on liver and kidney function | 🞎 | 🞎 | 🞎 |
| Drug contraindications for special diseases | 🞎 | 🞎 | 🞎 |

1. Please indicate the information you wanted in adverse drug reactions?

|  | Extremely needed | Moderately needed | No need |
| --- | --- | --- | --- |
| Clinical features | 🞎 | 🞎 | 🞎 |
| Treatment of adverse drug reactions | 🞎 | 🞎 | 🞎 |
| Treatment of drug overdoses | 🞎 | 🞎 | 🞎 |

1. Which of the following ways would you like to have a medication consultation?

🞎 Face-to-face consultation when getting the medicine

🞎 Internet consultation

🞎 Drug counseling clinic

🞎 Telephone consultation

1. Do you wish to receive a reminder service from the medication software?

🞎Yes

🞎 No

**Medication adherence**

1. Do you experience instances of missed doses or non-adherencee with medication instructions?

🞎 Frequently

🞎 Occasionally

🞎 Rarely

🞎 Never
