# Supplementary material for: Intelligent medication manager: developing and implementing a mobile application based on WeChat
Source: Front Pharmacol. 2023 Aug 21;14:1253770. doi: 10.3389/fphar.2023.1253770 (PMC10475577; doi:10.3389/fphar.2023.1253770)
Supplement: Supplementary file 2 [file Table2.DOCX]

**Questionnaire 2**

**Patient Characteristics**

1. Your gender

🞎 Male

🞎 Female

2. Your age

🞎 < 35 years old

🞎 35-49 years old

🞎 50-64 years old

🞎 ≥65 years old

3. Your marital status

🞎 Single

🞎 Married

🞎 Divorced

🞎 Other

4. Your educational status

🞎 High school or below

🞎 College degree or bachelor's degree

🞎 Master's degree or above

5. Where you live

🞎 Town

🞎 Country

1. Number of previous outpatient services at the present facility

🞎 One

🞎 Two

🞎 More than two

1. The department in which you were treated

🞎 Internal medicine department

🞎 Surgery department

🞎 Gynecology and obstetrics

🞎 Pediatric department

🞎 Psychiatry department

🞎 Other

**The system usability scale.**

1. Please indicate your level of recognition of the system usability scale.

|  | Extremely agree | Moderately agree | Disagree |
| --- | --- | --- | --- |
| I thought I would like to use this system frequently. | 🞎 | 🞎 | 🞎 |
| I thought the system was easy to use. | 🞎 | 🞎 | 🞎 |
| I thought I would need the support of a technical person to be able to use this system | 🞎 | 🞎 | 🞎 |
| I found that the various functions in this system were well integrated | 🞎 | 🞎 | 🞎 |
| I found the system unnecessarily complex | 🞎 | 🞎 | 🞎 |

**The demand and satisfaction of outpatients with the system.**

1. Please indicate the necessity with the XMG

🞎 Very necessary

🞎 Moderately necessary

🞎 Unnecessary

1. Please indicate your level of satisfaction with the XMG

🞎 Very satisfied

🞎 Moderately satisfied

🞎 Not at all satisfied

1. Do you think that XMG can meet your needs for medicine?

🞎 Yes

🞎 No

1. What is the maximum cost of the medication consultation you are willing to pay?

🞎 ¥ 21-50

🞎 ¥ 11-20

🞎 ¥ 1-10

🞎 ¥ 0

1. What needs to be improved or perfected in the XMG?

|  |
| --- |

**Medication compliance**

1. Do you experience instances of missed doses or non-compliance with medication instructions?

🞎 Frequently

🞎 Occasionally

🞎 Rarely

🞎 Never
