# Supplementary material for: Intelligent medication manager: developing and implementing a mobile application based on WeChat
Source: Front Pharmacol. 2023 Aug 21;14:1253770. doi: 10.3389/fphar.2023.1253770 (PMC10475577; doi:10.3389/fphar.2023.1253770)
Supplement: Supplementary file 3 [file Table5.docx]

**Supplemental Table 5**. Analysis of patient satisfaction and medication adherence among XMG users of surveyed (n=407)

| **Item** | **Number (percentage)** |
| --- | --- |
| Satisfied with XMG |  |
| Very satisfied | 372 (91.40%) |
| Generally satisfied | 32 (7.86%) |
| Not satisfied | 3 (0.74%) |
| Medication adherence level |  |
| Good | 228 (56.02%) |
| Poor | 179 (43.98%) |
