# Supplementary material for: Intelligent medication manager: developing and implementing a mobile application based on WeChat
Source: Front Pharmacol. 2023 Aug 21;14:1253770. doi: 10.3389/fphar.2023.1253770 (PMC10475577; doi:10.3389/fphar.2023.1253770)
Supplement: Supplementary file 4 [file Table3.DOCX]

**Supplemental Table 3**. The need and patient requirements for medication guidance (n=419).

| **Item** | **Number (proportion)** |
| --- | --- |
| The necessity of medication guidance |  |
| Very necessary | 369 (88.07%) |
| General necessary | 43 (10.26%) |
| No need | 7 (1.67%) |
| Ways to solve the drug confusion |  |
| Consult a doctor | 357 (85.20%) |
| Consult a pharmacist | 73 (17.42%) |
| Read the instructions | 127 (30.31%) |
| Others | 59 (14.08%) |
| Content preference |  |
| Indications | 271 (64.68%) |
| Dosage and administration | 305 (72.79%) |
| Contraindications and precautions | 275 (65.63%) |
| Adverse drug reactions | 219 (52.26%) |
| Drug-drug interaction | 133 (31.74%) |
| Storage | 109 (26.01%) |
| Expiration date | 79 (18.85%) |
| Format of medication guidance |  |
| Face to face with pharmacists | 263 (62.77%) |
| Internet consultation | 121 (28.88%) |
| Drug counseling clinic | 167 (39.86%) |
| Telephone consultation | 71 (16.95%) |
| Medication reminder service |  |
| Need | 328 (78.28%) |
| Don't need | 91 (21.72%) |
| Medication adherence level |  |
| Good | 198 (47.26%) |
| Poor | 221 (52.74%) |
