# Supplementary material for: Intelligent medication manager: developing and implementing a mobile application based on WeChat
Source: Front Pharmacol. 2023 Aug 21;14:1253770. doi: 10.3389/fphar.2023.1253770 (PMC10475577; doi:10.3389/fphar.2023.1253770)
Supplement: Supplementary file 5 [file Table4.DOCX]

**Supplemental Table 4**. Demographics of the sample in the second survey (n=407)

| **Variable** | **Number (proportion)** |
| --- | --- |
| *Sex* |  |
| Male | 208 (51.11%) |
| Female | 199 (48.89%) |
| *Age* |  |
| < 35 | 209 (51.35%) |
| 35-49 | 91 (22.36%) |
| 50-64 | 65 (15.97%) |
| ≥ 65 | 42 (10.32%) |
| *Marital status* |  |
| Married | 202 (49.63%) |
| Separated/divorced/widowed | 205 (50.37%) |
| *Educational background* |  |
| High school or below | 147 (36.12%) |
| University | 231 (56.76%) |
| Postgraduate or above | 29 (7.13%) |
| *Residence* |  |
| Urban | 253 (62.16%) |
| Rural area | 154 (37.84%) |
| *Number of medical visits* |  |
| 1 | 133 (32.68%) |
| 2 | 148 (36.36%) |
| ≥3 | 126 (30.96%) |
